# Phosphoproteomics Preliminarily Revealed the Differential Phosphorylation Sites and Their Functional Significance in the Longissimus Dorsi Muscle of Kazakh and Xinjiang Brown Cattle

**DOI:** 10.1002/fsn3.72037

**Published:** 2026-06-15

**Authors:** Ailifeire Wumaier, Zhen Ma, Wenzhong Chen, Meng Liu, Sumei Chen, Sihan Wang, Wenjing Wang, Xiao Wang, Xiangmin Yan, Lei Chen

**Affiliations:** ^1^ Institute of Animal Husbandry Xinjiang Academy of Animal Husbandry China; ^2^ College of Animal Science Xinjiang Agricultural University China; ^3^ Yili Vocational and Technical College China; ^4^ School of Animal Science and Technology Shihezi University Shihezi China

**Keywords:** Kazakh cattle, phosphoproteomics, RAB12, the longissimus dorsi muscle tissue, Xinjiang brown cattle

## Abstract

As the life science technology developed fast, various proteomics were applicable to human medical research and livestock production. To further study the differences in muscle production and development between Kazakh cattle and Xinjiang brown cattle, we performed phosphoproteomic analysis of the longissimus dorsi muscle tissue of Xinjiang brown cattle and Kazakh cattle. We found that there were 22 disparately expressed proteins, of which 6 were upregulated and 16 were downregulated. There were 28 differentially phosphorylated sites, including 6 sites with increased phosphorylation levels and 22 sites with decreased phosphorylation levels. In addition, PRM revealed that the levels of PDLIM3, PPP1R3A, MYPN, and IGFN1 were significantly increased in Xinjiang brown cattle, and these results were similar to those of the phosphoproteomic analysis. Based on the species conservation, we knocked down the expression of RAB12 in C2C12 cells. We found that after RAB12 was knocked down, the protein levels of the myogenic differentiation marker MYOG were significantly increased, and the immunofluorescence intensity of MYHC was also increased.

## Graphical abstract

The phosphorylated protein profiles of the longissimus dorsi muscle tissues of Xinjiang brown cattle and Kazakh cattle were analyzed. RAB12 was a differentially expressed phosphorylated protein. After knockdown of RAB12, the immunofluorescence intensity of MYHC significantly increased, promoting myoblast differentiation.

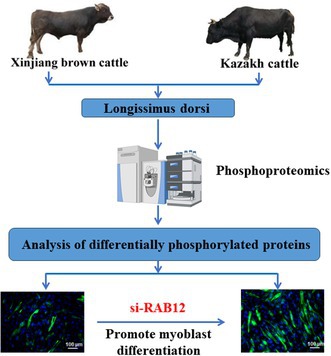

## 1 Introduction

The quality of beef is crucial to people's living standards, and it is affected by many factors, such as genetic, nutritional, and so on environmental factors (Sakowski et al. 2022). These factors not only affect the health and yield of the cattle, but also directly and indirectly impact the quality of the beef produced, including its taste, tenderness, juiciness, and nutritional value. Different cattle breeds have different meat qualities (Mwangi et al. 2019). Additionally, individual genetic variation within one cattle breed affects meat quality. Inherited traits affect muscle composition, fat distribution, and growth rate, which in turn affect the taste and texture of beef (Purslow et al. 2021). The overall quality of beef is often the result of a combination of factors. Meat quality is influenced by numerous factors, and identifying key regulatory proteins and modifications is a target for improvement, complementing strategies such as the use of natural enhancers (Abubaker et al. 2025), (Al‐Wraikat et al. 2024). Strategies targeting any of the related factors can improve beef quality, and farmers can fine‐tune these strategies to meet consumer preferences and industry standards.

Phosphorylation is a key type of posttranslational modification that can alter the function, activity, localization, or interactions of a protein. Phosphoproteomics is a widely used technique in animal studies. Phosphoproteomics can be applied to study phosphorylation events that regulate reproductive processes, such as ovulation, fertilization, and embryonic development, as well as the effects of phosphorylation on skeletal muscle structure and other aspects. For example, phosphorylation of myosin light chain affects the structure of thick myofilaments in rabbit skeletal muscle (Levine et al. 1996). Additionally, preventing phosphorylation of RyR2 can reduce calcium ion leakage and prevent cardiac muscle disorders by reactivating the INa current (Zheng et al. 2025). Moreover, the tyrosine phosphorylation process of the myosin heavy chain in both skeletal muscle and platelets may trigger reorganization of the cytoskeleton in muscle cells and platelets (Arrington et al. 2017; Blazev et al. 2022; Harney et al. 2005; Zeng et al. 2022). Phosphoproteomics could be used to study phosphorylation events that affected traits like growth rate, meat quality, milk yield, and disease resistance in livestock (Xin et al. 2017). Label‐free phosphoproteomic technology was used to analyze colostrum and mature milk in cattle, and it was found that perilipin‐2 had the highest number of phosphorylation sites in mature bovine milk; notably, MFGM phosphoproteins in bovine colostrum are involved mainly in immune functions, while those in mature bovine milk are associated primarily with metabolic functions (Bai et al. 2024). Quantitative phosphoproteomic methods have been utilized to elucidate the mechanisms of muscle fiber development in goslings, with a total of 6412 phosphorylation sites quantified, corresponding to 5548 phosphopeptides and 2519 phosphoproteins; compared with goslings with high meat production and low fiber density, goslings with high meat production and high fiber density showed higher phosphorylation levels of muscle structural proteins, cytoskeletal proteins, and components of the MAPK signaling pathway (Weng et al. 2026). Phosphoproteomic analysis of the pectoral muscles of Qingyuan Partridge chickens and Cobb broilers revealed that the phosphorylation of PPP1R3A regulates glycogen metabolism, thereby affecting the content of precursors for the Maillard reaction (Yang et al. 2026). Quantitative phosphoproteomic analysis of the longissimus thoracis, semimembranosus, iliopsoas, and semitendinosus muscles of pigs revealed 13,232 phosphopeptides from 3137 phosphoproteins, and protein–protein interaction networks demonstrated that phosphoproteins regulate differences in pork quality (Zhang et al. 2025). Furthermore, proteomic and phosphoproteomic analyses of beef during post mortem aging revealed 9 common differentially expressed proteins (DEPs); pathway analysis indicated that selenium compound metabolism, tryptophan metabolism, and cysteine and methionine metabolism are involved in flavor formation during wet aging of beef after ultrasonic treatment (Wang et al. 2024).

Kazakh cattle are a dual‐purpose animal that can produce both milk and meat (Li et al. 2020). Kazakh cattle are known for their endurance and ability to survive extreme weather conditions. They usually live in steppe and semidesert areas, which shows their adaptability to different environments. Their value lies in their high‐quality meat and ability to produce milk with a high milk fat content (Chen et al. 2022). Xinjiang brown cattle are a cross between Kazakh brown cattle and Switzerland brown cattle (Yan et al. 2021). Xinjiang brown cattle are mainly produced in the Xinjiang Uygur Autonomous Region of China. This breed is known for its robustness and adaptability to harsh climates. The main products of Xinjiang brown cattle are meat and milk, and their ability to produce high‐quality beef is particularly prominent. This variety adapts well to the high‐altitude environment, making it suitable for living in the vast grasslands of the region. Xinjiang brown cattle and Kazakh cattle have unique characteristics and environmental adaptations. In a previous article, we explore the differentially expressed proteins in the longissimus dorsi muscle tissues of Xinjiang brown cattle and Kazakh cattle and report that 75 proteins are upregulated and 44 proteins are downregulated (Ma et al. 2023). However, the specific phosphorylation site is unknown and needs to be further investigated.

In this study, phosphoproteomics was used to identify the phosphorylation sites of proteins in the longissimus dorsi muscle tissues of Kazakh cattle and Xinjiang brown cattle. We screened differentially expressed proteins and phosphorylation sites in Xinjiang brown cattle and Kazakh cattle. On the basis of species conservation, we knocked down the expression level of RAB12 in C2C12 cells. We found that knocking down RAB12 promotes myoblast differentiation. This study will provide a theoretical basis for the improvement of beef quality and the cultivation of new varieties.

## 2 Materials and Methods

### 2.1 Ethics Statement

The experiments were carried out in strict accordance with the guiding principles of the Guide to Laboratory Animal Care and Use of the Xinjiang Academy of Animal Science. In addition, the entire experimental procedure was approved by the Institutional Animal Care and Use Committee of Xinjiang Academy of Animal Science (license number: 2024001).

### 2.2 Sample Collection and Protein Extraction

Xinjiang brown cattle and Kazakh cattle were provided by the Xinjiang Yili Yixin Cattle and Sheep Breeding Cooperative. After slaughter, longissimus dorsi muscle tissue samples were collected immediately and placed into liquid nitrogen. Next, the samples were promptly sent to the company for tissue sample extraction. A total of 6 samples were tested, including 3 from Xinjiang brown cattle and 3 from Kazakh cattle. A total of 100 mg of tissue sample was weighed in a mortar that was precooled with liquid nitrogen. Next, liquid nitrogen was added, and the samples were ground completely into powder. Lysate was increased to each group of samples and ultrasonicated for cleaving. Then, it was taken to centrifuge for 10 min, at 4°C, 12000 g, and then using the BCA kit (Beyotime Biotechnology, Shanghai, China) to determine the protein concentration. Equal amounts of protein (100 μg per sample) were enzymatically hydrolyzed. Bovine serum albumin (BSA) standard protein (10 μg per sample) was added, and the volume was adjusted to 100 μL with TEAB buffer to match the lysate. Afterward, the mixture was vortexed and mixed; after 1 volume of precooled acetone was added, the precooled acetone was added four times and deposited at −20°C, and the process was continued for 2 h. To discard the supernatant, set at 4500 g, centrifuge for 5 min, and use pre‐cooled acetone to wash the pellet twice. After air‐drying, the pellet was resuspended in triethylammonium bicarbonate (TEAB) buffer at a final concentration of 200 mM, and further fragmented by sonication. Trypsin (Sigma‐Aldrich, USA) was added at a 1:50 (w/w) trypsin‐to‐protein ratio, and enzymatic hydrolysis was performed overnight at 37°C. Dithiothreitol (5 mM) was added, and the temperature was reduced to 56°C for 30 min. Add IAA to 11 mM and hatched it at a dark room thermal for 15 min to alkylate cysteine residues.

### 2.3 TMT Marker and Modification Enrichment

Strata X C18 (Phenomenex) was used to desalt the digested tryptic peptides, which were then vacuum freeze‐dried. The operations are as follows: First, unfrozen reagent and acetonitrile (0.1% formic acid in ACN, v/v) were used to dissolve the lyophilized peptide. The reconstituted peptide solution was mixed thoroughly with the pre‐equilibrated Strata X C18 column and incubated at 4°C for 2 h. The column was subsequently washed with 0.1% formic acid in ultrapure water to remove impurities, and the bound peptides were eluted with elution buffer, freeze‐dried in vacuum, and graded the peptides. The peptide fraction was subsequently merged into 4 components, and the merged components were freeze‐dried under vacuum for subsequent operations. The peptides in loading buffer, and the pre‐washed IMAC material (IMAC Sepharose 6 Fast Flow, GE Healthcare/Cytiva, Uppsala, Sweden) were used to divert the supernatant, finally placing the product on a shaker. Afterward, the material was washed three times with washing buffer. Finally, the phosphopeptides were eluted, and the product was dried under a vacuum. After the samples were dry, desalting.

### 2.4 Liquid Chromatography‐Mass Spectrometry Analysis

Peptides were dissolved in liquid chromatography mobile phase A (2% acetonitrile and 0.1% formic acid in ultrapure water) and separated. Liquid A was 2% acetonitrile and 0.1% formic acid in water, Liquid B was 90% acetonitrile and 0.1% formic acid in water. The peptide solution was injected into the liquid chromatography system for separation, and then injected into an NSI ion source for ionization after separation. The peptide precursor ions were subsequently detected and their secondary fragments were analyzed using a high‐resolution Orbitrap (Thermo Fisher Scientific). The first‐order mass spectrometry scanning range was 350–1400 m/z.

### 2.5 Differential Phosphoproteomic Analysis

We used MaxQuant (v2.7.3.0) in this experiment. The database is Bos_taurus_9913_PR_20220114.fasta, and we used a common contamination library to determine the effects of the polluting proteins. Trypsin/P was used as the digestion method. The length of the peptide was set to 7, and the number of peptide modifications was set to five. The mass error tolerance for primary and primary precursors was 20 and 4.5 ppm, respectively, while the mass error tolerance of the secondary fragment ions was 20 ppm. Set as a fixed modification, cysteine alkylation carbamidomethyl was identified as acetylation and deamidation of the N‐terminus of proteins. Variable modifications included phosphorylation on serine, threonine, and tyrosine residues. The ratio of the mean value of the relative quantification of the modification site in the replicate sample was used as a multiple of the difference. We also used *t*‐test to test the comparison group, and calculated the *p* value, and the default *p*‐value<was 0.05. Only sites with a localization probability > 0.75 were used for subsequent quantitative analysis to conform. On purpose of conforming the data of the *t*‐test requirements. Prior to testing, the relative modification site date underwent Log2 transformation.

### 2.6 Enrichment Analysis of Differentially Phosphorylated Proteins

To thoroughly understand the functions of the differentially phosphorylated proteins, we performed GO (Gene Ontology) classification and KEGG (Kyoto Encyclopedia of Genes and Genomes) pathway enrichment analyses of the differentially phosphorylated proteins in each comparison group using Metascape (v3.5.0, GNF) software. Gene Ontology enrichment analysis is a bioinformatics analysis method used to annotate genes and their products. GO annotation is classified into 3 categories: biological processes, cellular components, and molecular functions. We performed enrichment analyses of differentially phosphorylated proteins for each of the three categories in the GO classification.

### 2.7 C2C12 Cell Culture and Transfection

C2C12 cells were cultured in a humidified incubator at 37°C with 5% CO_2_. The growth medium consisted of DMEM (Vivacell, Shanghai, China) supplemented with 10% FBS (Lonser, Beijing, China) and 1% penicillin/streptomycin (Gibco, Grand Island, NY, USA). C2C12 cells at 70%–80% confluence were transfected with si‐RAB12 (GenePharma, Shanghai, China). We aimed to knock down RAB12 expression and further investigated its effect on C2C12 cell differentiation.

### 2.8 Western Blot Analysis

Western blot analysis was carried out according to the methods in the published literature. The tissues or cells were homogenized in protein lysate. Afterward, the samples were centrifuged for 10 min to extract the supernatant. After denaturation, the proteins were separated by SDS‐PAGE and transferred to a PVDF membrane (Millipore). The primary antibodies used were an anti‐phospho‐RAB12 (S106) rabbit monoclonal antibody (ab256487, Abcam) diluted at 1:1000, anti‐RAB12 rabbit monoclonal antibody (ab316770, Abcam) diluted at 1:1000, anti‐MYOG mouse monoclonal antibody (DF8273, Affinity) diluted at 1:500, and anti‐Tubulin rabbit polyclonal antibody (Abcam) diluted at 1:5000. The membrane was then washed with TBST three times and incubated with horseradish peroxidase (HRP)‐labeled secondary antibody (Abcam) at room temperature for 1 h. Protein imaging was performed using chemiluminescence, and band intensities were quantified using ImageJ software for relative expression analysis.

### 2.9 Immunofluorescence

After the culture medium was removed, the cells were rinsed with PBS solution and fixed with 4% paraformaldehyde for 15 min. The cell membranes were permeabilized using Triton X‐100 (Beyotime, Shanghai, China) for 10 min, and then blocked with 5% BSA (Solarbio, Beijing, China) for 1 h at room temperature to prevent nonspecific binding. The cells were incubated with anti‐MYHC antibody (Santa Cruz Biotechnology, Dallas, TX, USA) at 1:200 in 1% BSA overnight at 4°C. The cells were then washed three times with PBS. The cells were incubated with a 1:500 dilution of secondary antibody (Epizyme Biotechnology, Shanghai, China) in the dark for 1 h. The sections were restained with a 1:1000 dilution of DAPI (Beyotime, Shanghai, China) at room temperature for 5 min. The fluorescence images were taken using a laser confocal scanning microscope (Leica, LSM 880 NLO).

### 2.10 Statistical Analysis

The data are presented as the mean ± SEM. In the GraphPad Prism 10.4.1 software, a *t*‐test was used to conduct statistical analysis on the two sets of data. A *p* < 0.05 was considered statistically significant.

## 3 Results

### 3.1 Phosphorylated Proteomic Sequencing Analysis of Longissimus Dorsi Muscle Tissue From Kazakh Cattle and Xinjiang Brown Cattle

**FIGURE 1:**
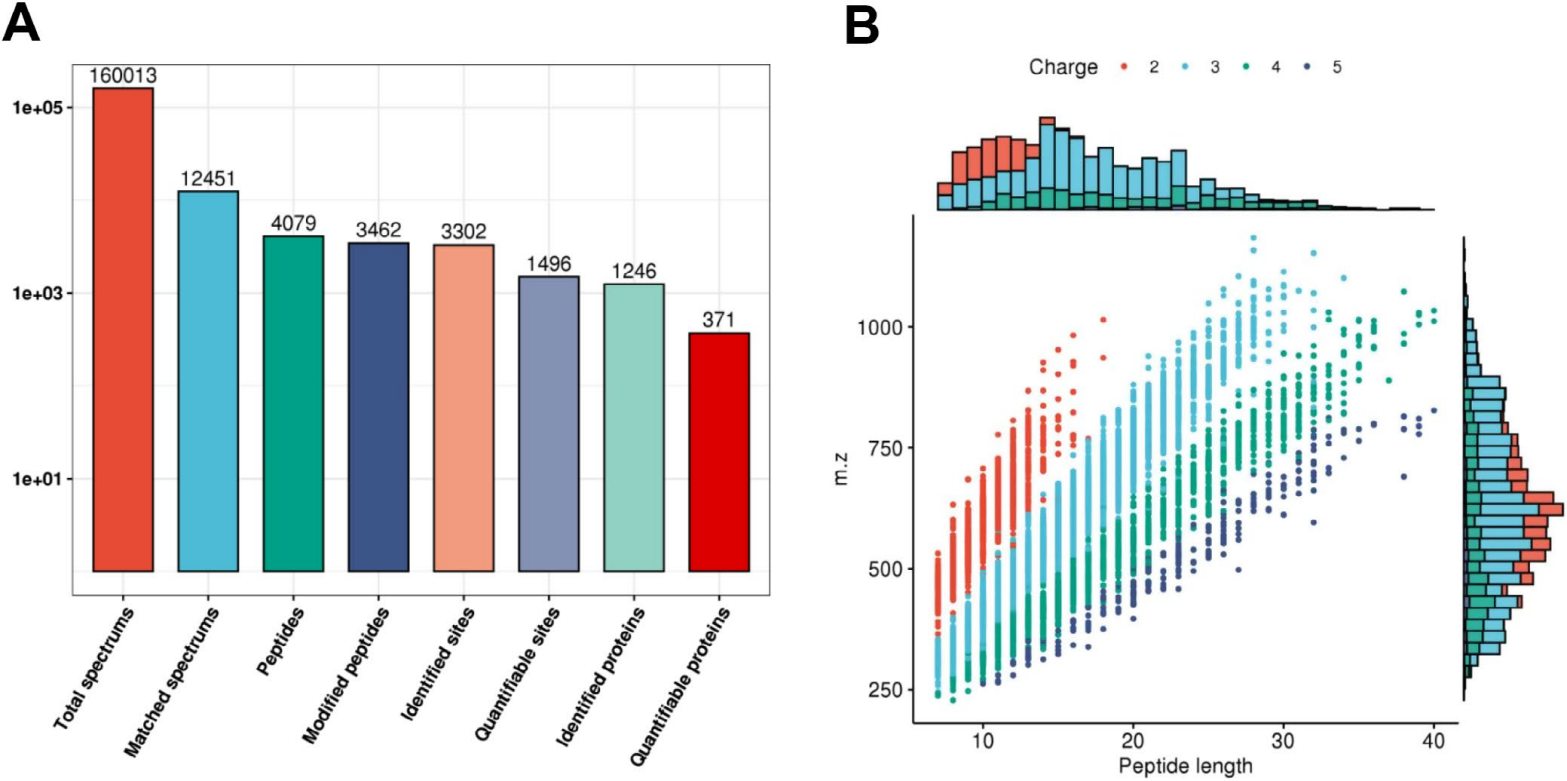
Phosphoproteomic sequencing analysis of longissimus dorsi muscle tissue from Xinjiang brown cattle and Kazakh cattle. (A) The overall quantity of peptides, modified peptides, phosphorylation sites and proteins identified after data filtering. (B) Conducting a quality control evaluation of the data under the mass spectrometry machine, showing the peptide length distribution, charge state (color‐coded for 2+ to 5+), and *m*/*z* ratio to ensure that the quality of the results meets the standards.

We extracted proteins from the longissimus dorsi muscle tissues of Kazakh cattle and Xinjiang brown cattle for phosphoproteomic sequencing analysis. To obtain high‐quality results, we needed to further filter the results of the search. The identified proteins needed to have at least one of the specific peptides. A total of 160,013 spectra were obtained from the filtered phosphoproteomics dataset, of which 12,451 were matched with peptide sequences (Figure 1A). This resulted in the identification of 4079 peptides, of which 3462 were modified peptides. In total, 1246 proteins were identified, of which 371 proteins were reliably quantified. In addition, 3302 phosphorylation sites were identified, and of these 1496 were quantifiable. It was necessary to perform a quality control evaluation on the data from the mass spectrometry instrument to ensure that the quality of the results meets the standard, including the peptide length distribution. Most of the peptides were distributed between 7 and 20 amino acids, based on enzymatic digestion and mass spectrometry fragmentation (Figure 1B). We used mass spectrometry to determine whether the distribution of peptide lengths could reach the QC demanded.

### 3.2 Screening and Analysis of Phosphorylation Modification Sites for Differential Expression

**FIGURE 2:**
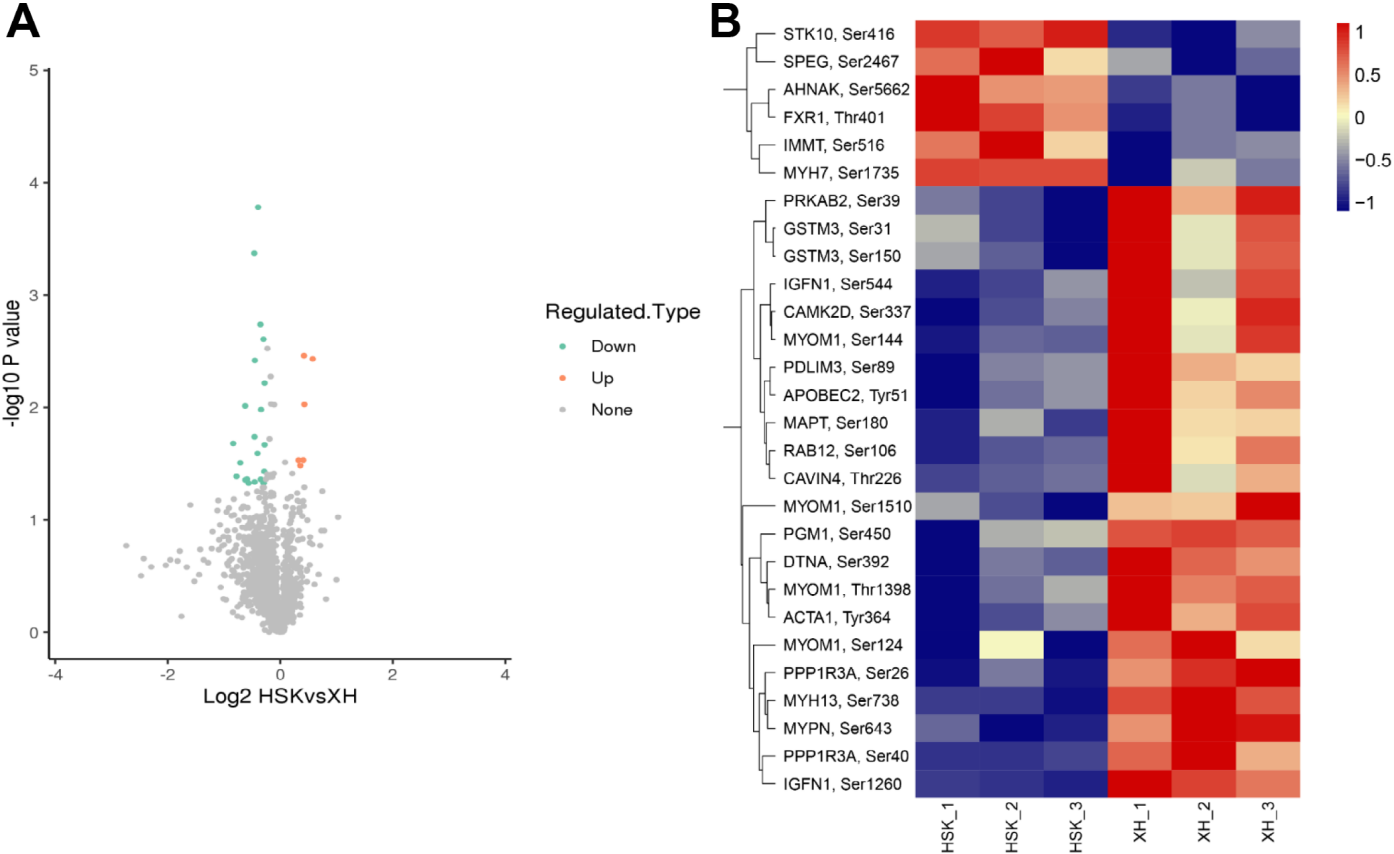
Screening and analysis of phosphorylation sites for differential expression. (A) Volcano plot of differential protein expression in the Kazakh cattle (HSK) and Xinjiang brown cattle (XH) phosphorylation groups. Showing log_2_ fold change (HSK vs. XH) vs. −log_10_(*p‐*value); up‐regulated (orange), down‐regulated (green), and non‐significant (gray) sites are defined by fold change > 1.2 or < 0.83 and *p* < 0.05. (B) Unsupervised clustering of differentially expressed proteins in the phosphoproteome.

We could get the percentage of the mean of the quantification values, and the mean was used to calculate the modification sites in multiple replicates. The percentage was adopted as a multiple of the difference. To determine the implications of the difference, we tested the relative degree of the value of each modification site in the comparative group by *t*‐test, and the related *p* value was extrapolated as a significance index, and the default *p* value was < 0.05. When the *p* value was < 0.05, the transform of differential expression amount exceeded 1.2 as the change threshold for significance increased. The volcano diagram of the differentially expressed modification sites is shown in Figure 2A, and the results showed that there are 22 differentially expressed proteins, of which 6 were upregulated and 16 were downregulated. There were 28 diverse modification sites, including 6 upregulated differential sites and 22 downregulated differential proteins. Figure 2B presents a heat map of the phosphorylation modification sites for differential expression.

### 3.3 Motif Analysis of Phosphorylation Sites

**FIGURE 3:**
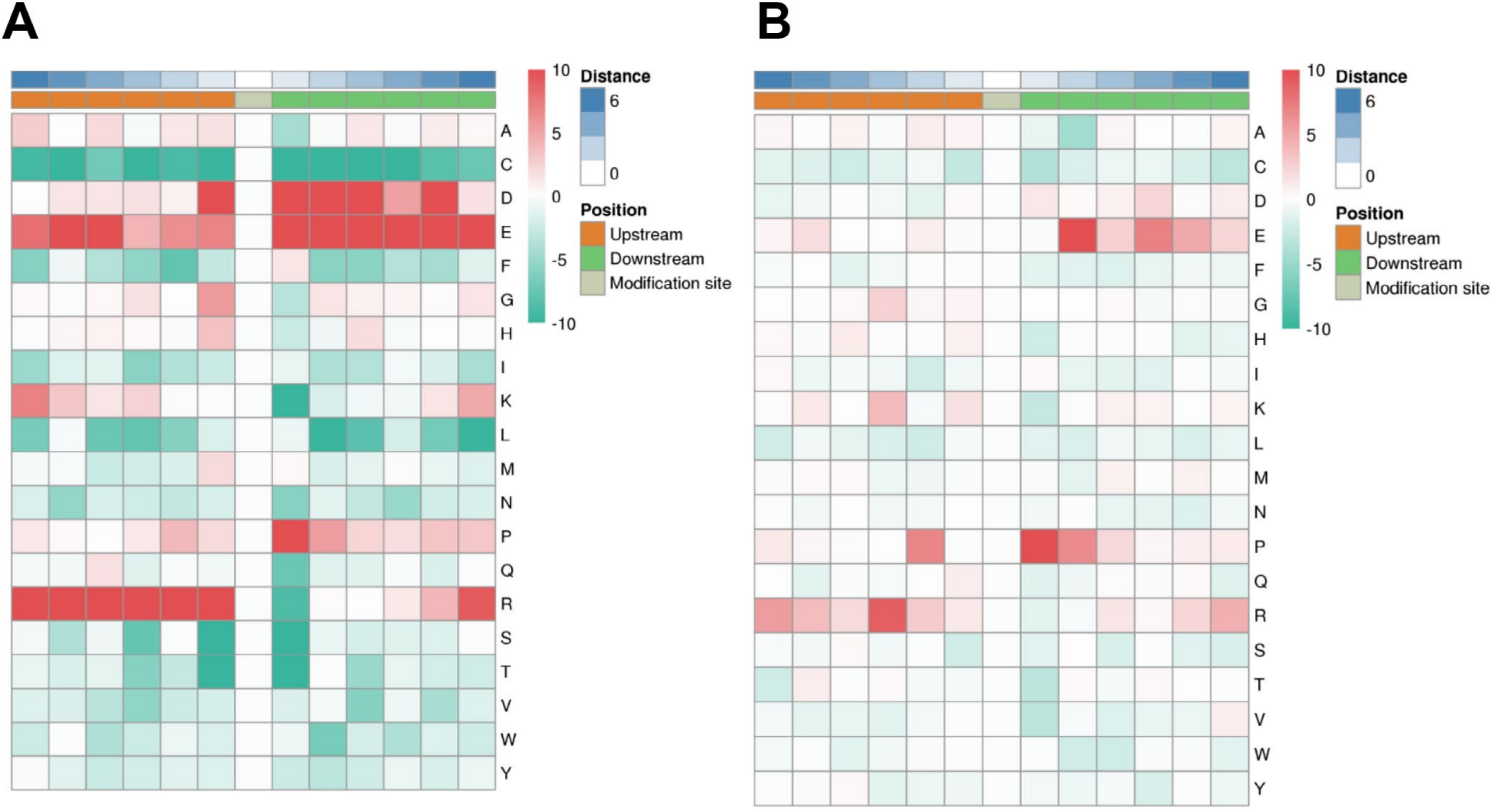
Motif analysis of phosphorylation sites. Heatmap excluding motif enrichment of amino acids downstream and upstream of all identified S (A) and T (B) modification sites. Red indicates a significant decrease in the concentration of the amino acid close to the modification site, and green indicates a significant decrease in the concentration of the amino acid close to the modification site.

A total of 26 phosphorylation motifs were recognized, including 22 pSer motifs and 4 pThr motifs (Table S1), which were analyzed using MoMo software based on the motif‐x algorithm. The first four positions in the pSer motif were [xxxxxx_S_DxEExx], [xxxxxx_S_ExEExx], [xxxRxx_S_PxPxxx], and [xxxxxx_S_EEExxx]. The four positions in the pThr motif are [xxxxPx_T_Pxxxxx], [xxxxxx_T_PPxxxx], [xxxxxx_T_Pxxxxx], and [xxxxxx_T_xExxxx]. The amino acid heatmap around the phosphorylation site indicated obvious concentrations of aspartic acid (D), arginine (R), and glutamic acid (E) in the identified S and T residues (Figure 3A,B). Around the identified S and T residues, the presence of cysteine (C), phenylalanine (F), and leucine (L) was significantly reduced (Figure 3A,B).

### 3.4 Enrichment Analysis of Differentially Modified Proteins

**FIGURE 4:**
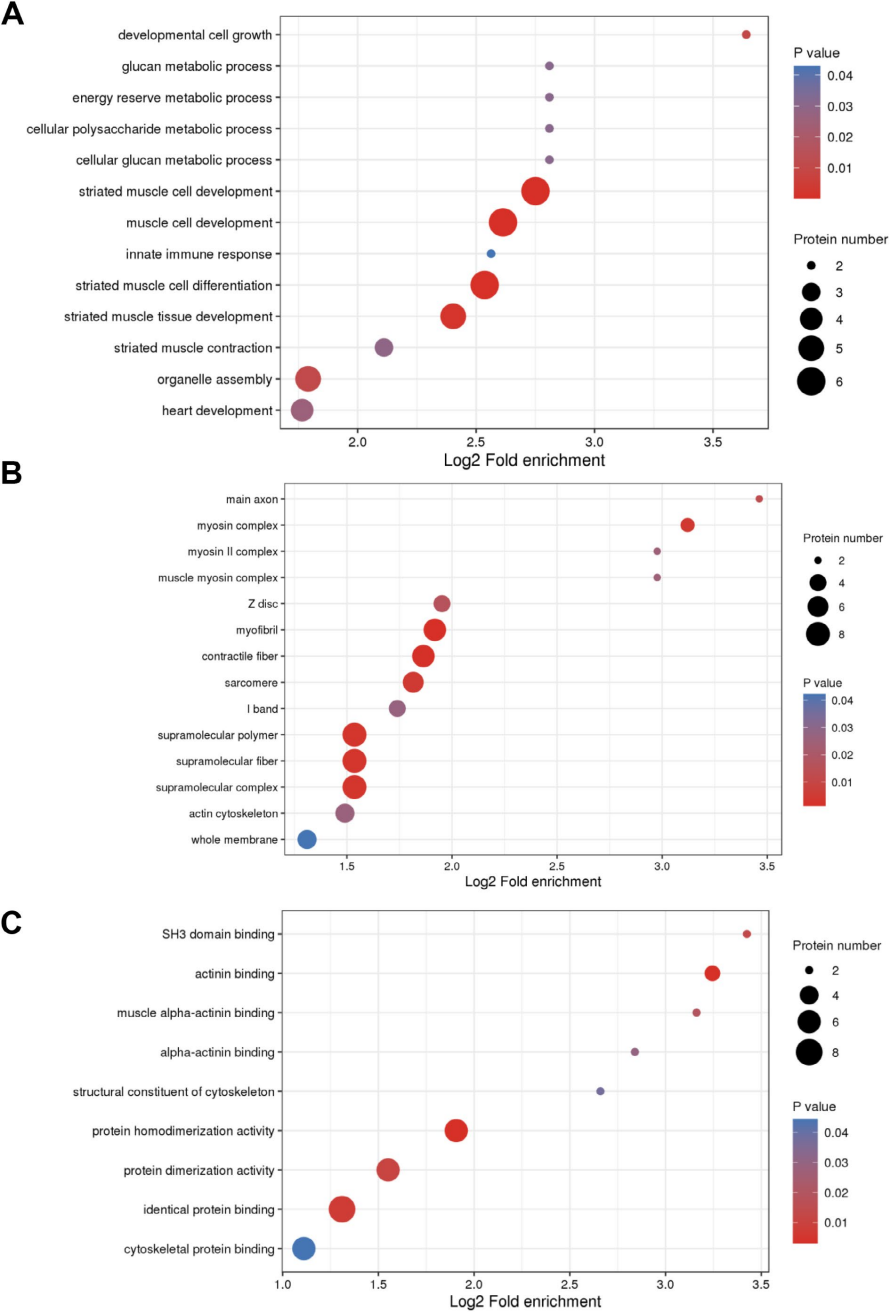
Functional enrichment analysis of phosphorylation sites for differential expression. Proteins were categorized to (A–C) biological process, (B) cellular component and (C) molecular function categories. *X*‐axis: log_2_‐transformed fold enrichment of each term; *Y*‐axis: Enriched functional terms.

Biological processes such as striated muscle cell development, developmental cell growth, and striated muscle cell differentiation were significantly enriched (Figure 4A). Cellular component contained myosin complex, myofibril, and contractile fiber significant enrichment (Figure 4B). The enriched molecular functions included SH3 domain binding, actinin binding, and protein homodimerization activity (Figure 4C).

### 3.5 PRM For Identifying Differentially Phosphorylated Proteins

**FIGURE 5:**
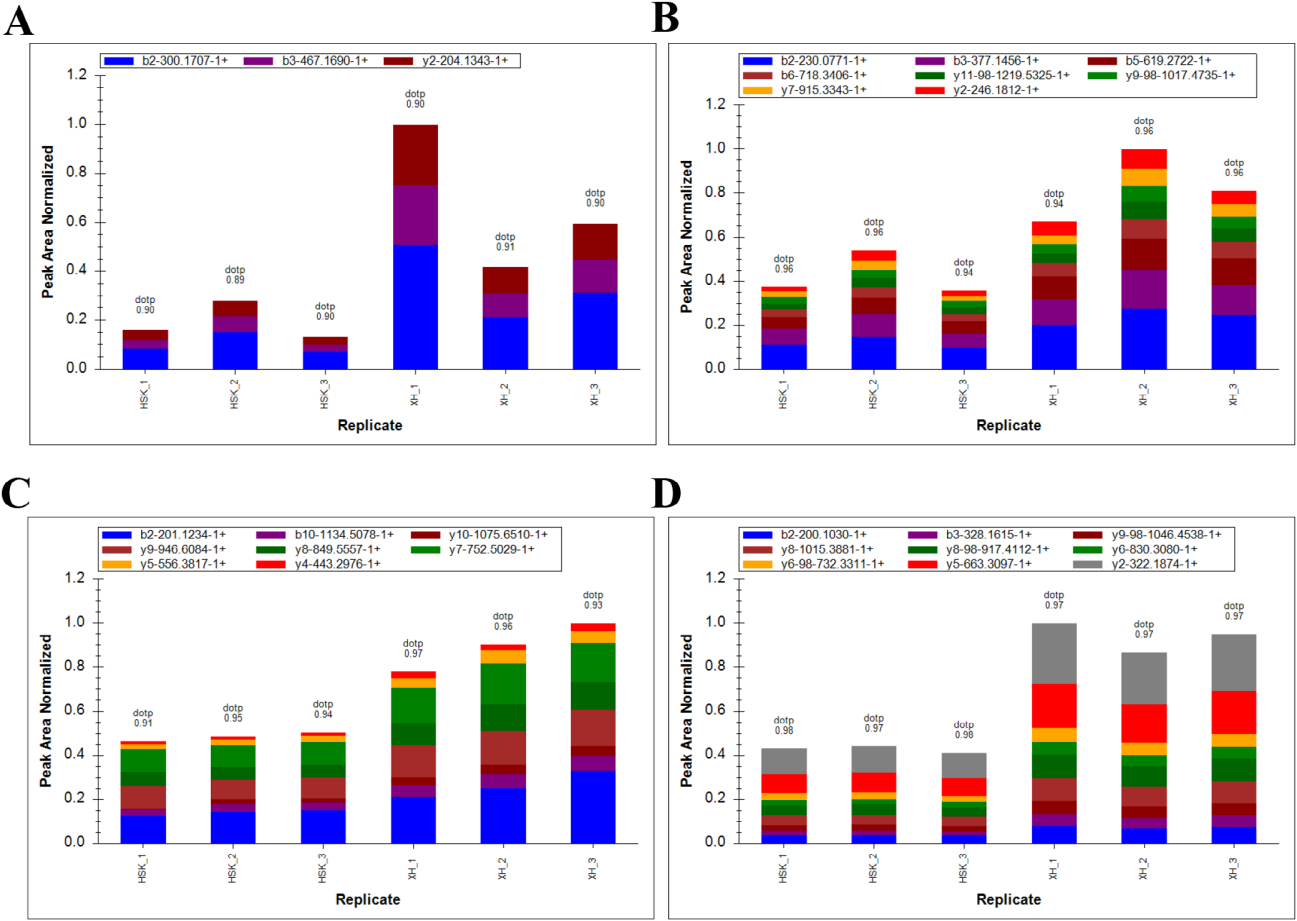
PRM for identifying differentially phosphorylated proteins. (A–D) The distribution of fragment ion peak areas of (A) PDLIM3, (B) PPP1R3A, (C) MYPN, and (D) IGFN1. PRM was quantified using peak area.

In this project experiment, we performed PRM quantification of the selected target modified peptide segments in samples from Xinjiang brown cattle and Kazakh cattle. Owing to the characteristics of the peptide segments and their expression levels, we quantified the modified peptide segments of PDLIM3, PPP1R3A, MYPN, and IGFN1. The results showed that the peak areas of PDLIM3 (Figure 5A), PPP1R3A (Figure 5B), MYPN (Figure 5C), and IGFN1 (Figure 5D) in Xinjiang brown cattle significantly increased. The trend of the results was similar to that of the phosphoproteomic results, which confirmed the reliability of our results.

### 3.6 Knocking Down RAB12 Promotes Myoblast Differentiation

**FIGURE 6:**
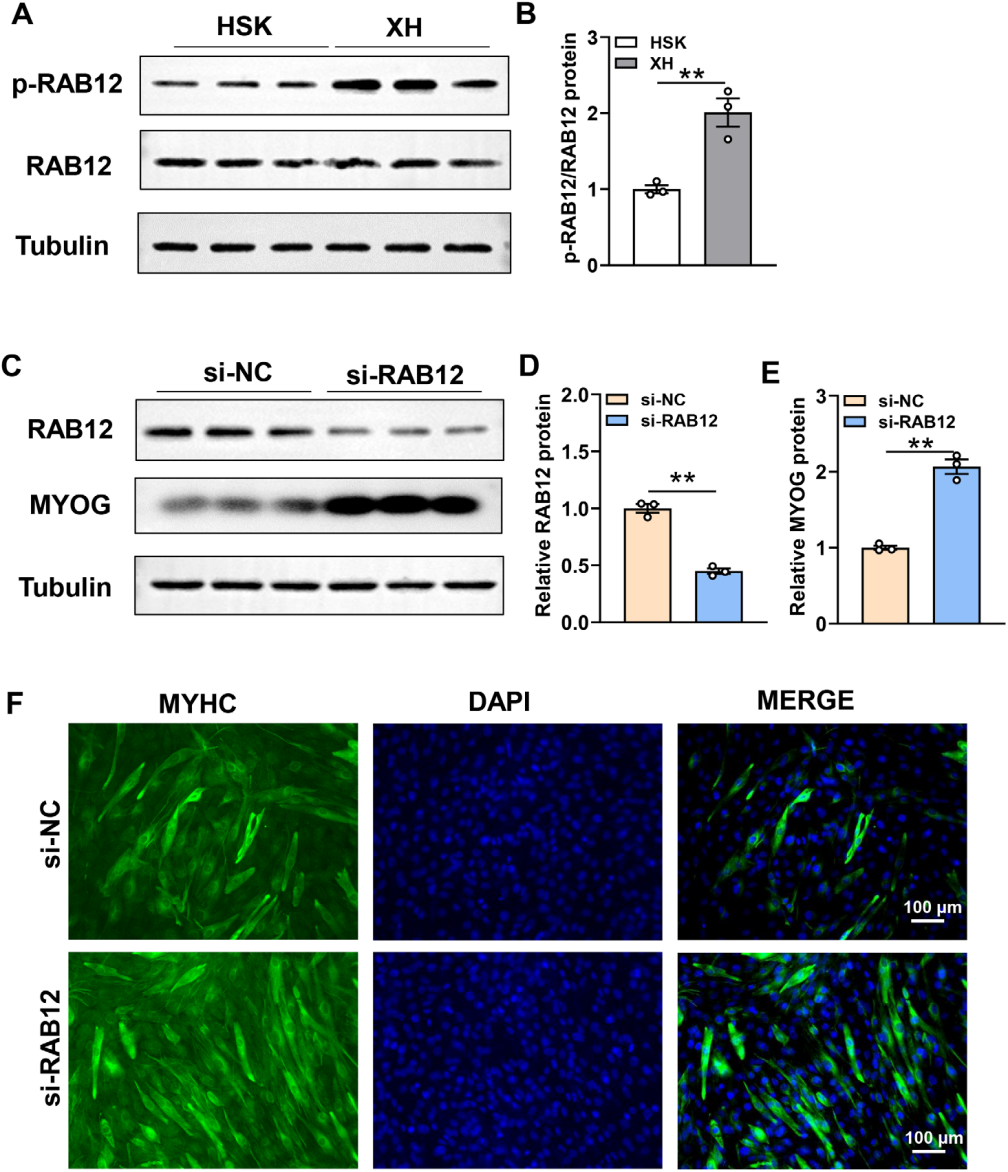
RAB12 knockdown promotes myoblast differentiation. (A, B) Phosphorylation level of RAB12 in Xinjiang brown cattle and Kazakh cattle. (C) After C2C12 cells were transfected with si‐RAB12, the protein level of RAB12 and the protein level of MYOG were measured. (D) Quantitative results of the protein level of RAB12. (E) Quantitative results of the protein level of MYOG. (F) After C2C12 cells were transfected with si‐RAB12, the fluorescence intensity of MYHC was measured.

On the basis of a literature review, we found that the Ser106 site of RAB12 is conserved across species. Therefore, we wanted to investigate whether RAB12 affects myoblast differentiation. We evaluated the phosphorylation levels of RAB12 in Xinjiang brown cattle and Kazakh cattle. The results revealed that the level of RAB12 phosphorylation was significantly greater in Xinjiang brown cattle than in Kazakh cattle (Figure 6A,B). On the basis of the species conservation of RAB12, we knocked down RAB12 expression in C2C12 cells and investigated the effect of RAB12 knockdown on myoblast differentiation. After RAB12 was knocked down, the protein levels of the myoblast differentiation marker MYOG significantly increased (Figure 6C,E), and the immunofluorescence level of MYHC significantly increased (Figure 6F). In conclusion, our experiments revealed that RAB12 knockdown promoted myoblast differentiation.

## 4 Discussion

Phosphoproteomics can be used to identify specific phosphorylation patterns associated with animal diseases, such as cancer, infectious diseases, and metabolic disorders (Thingholm et al. 2009). Phosphoproteomics are a powerful tool to deepen our understanding of animal biology, improve disease diagnosis and treatment, improve livestock production, and support the development of new therapies (Chan et al. 2016; Higgins et al. 2023; Moonmuang et al. 2023). Phosphoproteomics can reveal signaling pathways that are activated under stress conditions, leading to better management methods (Macek et al. 2009). The study of phosphorylated proteins involved in metabolic pathways can provide insight into how different diets affected the health and performance of animals. This information is essential for developing diets that optimize growth, reproduction, and health (Kreuzer et al. 2019). In this study, phosphoproteomics was used to determine the phosphorylation sites of proteins in the longissimus dorsi muscle tissues of Xinjiang brown cattle and Kazakh cattle. This study facilitated to the in‐depth analysis of the differences between the longissimus dorsi muscle tissue of Xinjiang brown cattle and Kazakh cattle, and provided a theoretical basis for the improvement of beef quality and the cultivation of new breeds.

The PDZ and LIM Domain 3 (PDLIM3) was a large protein family. Members of this family included a PDZ domain at the N‐terminus and a LIM domain at the C‐terminus (Liu and Fuentes 2019). The absence of PDLIM3 significantly hindered the formation of cilia by inhibiting the Hh signaling pathway. The PDLIM3 protein might physically interact with cholesterol to promote ciliogenesis (Zhang et al. 2023). In the muscle tissues of mice transfected with a mutant of PPP1R3A, the key regulator of muscle glycogen metabolism, the truncated mutant was unable to bind glycogen, thereby decreasing glycogen synthase activity and increasing the activity of glycogen phosphorylase (Savage et al. 2008). A previous study revealed that PPP1R3A expression tended to be downregulated in the atria of patients with paroxysmal and persistent atrial fibrillation. The decrease in the expression of the PPP1R3A gene weakened the targeting of PP1 while increasing the levels of RyR2 and PLN phosphorylation. In this study, we found that PDLIM3 and PPP1R3A were significantly upregulated and might be important regulatory factors for muscle differentiation.

Myopalladin protein (MYPN) was a multifunctional protein that could maintain the integrity of muscle fibers. A study had identified a SNP (A1795G located in exon 9) in the MYPN gene (Jiao et al. 2010). This SNP was found to have a significant effect on the ultrasonic area of the abdominal muscles in 399 individuals. This SNP might become a genetic marker for meat quality traits in cattle breeding and genetics. Through PCR‐RFLP technology, a SNP was identified in the 3'UTR region of the MYPN gene. This SNP was revealed to be related to the meat quality of Italian Large White pigs and Italian Duroc pigs (Braglia et al. 2013). Multiple proteins can be produced during protein synthesis of the IGFN1 gene through alternative splicing in skeletal muscle. The fusion index of the Igfn1‐deficient cell line isolated from C2C12 cells was reduced. Additionally, in the IGFN1‐deficient C2C12 cells during differentiation, the G:F actin ratio was significantly higher. Igfn1 interacted with COBL and stabilized its structure, preventing COBL from forming actin folds (Cracknell et al. 2020). Reducing the expression of IGFN1_v1 completely blocked myoblast fusion. In the Igfn1 exon 13 knockout cell line, the expression of IGFN1_v1 partially restored fusion and myotube morphology. However, overexpression of IGFN1_v1 or the CRISPR/Cas9 targeting vector of Igfn1 exon 13 in vivo did not change the size of the fibers (Li et al. 2017). In this study, we found that MYPN and IGFN1 were significantly upregulated and might be important regulatory factors for muscle differentiation. Small GTPase Rab family proteins are key regulatory factors for membrane transport in living organisms. RAB12 affected the PCBP1 expression through autophagy, thereby regulating glucose uptake in MSCs and subsequently controlling osteogenic differentiation (Ji et al. 2025). Research has shown that Rab12 was a novel autophagy regulatory factor that could control the degradation process of PAT4. Knockout of Rab12 might inhibit the autophagy process by increasing the amino acid uptake of PAT4 and activate the mTORC1 signaling pathway (Matsui and Fukuda 2013). RAB12 directly interacted with LRRK2 to recruit RILPL1, thereby inhibiting the generation of primary cilia in astrocytes (Li et al. 2024). The binding of RAB12 to the sites within the LRRK2 domain would activate LRRK2 to carry out Rab phosphorylation (Dhekne et al. 2023). In our study, the level of RAB12 protein phosphorylation was significantly greater in Xinjiang brown cattle than in Kazakh cattle. Given the conservation of RAB12 across species, we transfected si‐RAB12 into C2C12 cells. After RAB12 was knocked down, the protein levels of the myogenic differentiation marker MYOG significantly increased, and the immunofluorescence level of MYHC also significantly increased. Our experiments revealed that the deletion of RAB12 promoted the differentiation of myoblasts.

## 5 Conclusions

In this study, phosphoproteomics was used to identify the phosphorylation sites of proteins in longissimus dorsi tissues of Xinjiang brown cattle and Kazakh cattle. We screened 22 differentially expressed proteins and 28 phosphorylation sites between Xinjiang brown cattle and Kazakh cattle and utilized PRM technology to demonstrate that the changes in PDLIM3, PPP1R3A, MYPN, and IGFN1 levels were similar to those detected in the phosphorylated proteome. We transfected C2C12 cells with si‐RAB12 and determined that the absence of RAB12 promoted the differentiation of myoblasts. Taken together, the results of this study provide a theoretical basis for improving beef quality and the breeding of new varieties of cattle.

## Author Contributions

**Ailifeire Wumaier:** conceptualization, writing – review and editing. **Zhen Ma:** writing – original draft, methodology. **Wenzhong Chen:** software, project administration. **Meng Liu:** data curation, resources. **Sumei Chen:** investigation. **Sihan Wang:** validation. **Wenjing Wang:** formal analysis. **Xiao Wang:** supervision. **Xiangmin Yan:** funding acquisition, writing – review and editing. **Lei Chen:** visualization, writing – review and editing.

## Funding

This research was funded by Xinjiang Uygur Autonomous Region Science and Technology Major Project (No. 2022A02001‐1) and Xinjiang Uygur Autonomous Region Beef Cattle Industry Technology System (No. XJARS‐10‐04).

## Conflicts of Interest

The authors declare no conflicts of interest.

## Supporting information


**Table S1:** Phosphorylation characteristics of longissimus dorsi muscle tissue of Kazakh.

## Data Availability

The data that support the findings of this study are available from the corresponding author upon reasonable request.
